# Effect of Fasting on the Metabolic Response of Liver to Experimental Burn Injury

**DOI:** 10.1371/journal.pone.0054825

**Published:** 2013-02-05

**Authors:** Mehmet A. Orman, Marianthi G. Ierapetritou, Ioannis P. Androulakis, Francois Berthiaume

**Affiliations:** 1 Department of Chemical and Biochemical Engineering, Rutgers, The State University of New Jersey, Piscataway, New Jersey, United States of America; 2 Department of Biomedical Engineering, Rutgers, The State University of New Jersey, Piscataway, New Jersey, United States of America; Michigan State University, United States of America

## Abstract

Liver metabolism is altered after systemic injuries such as burns and trauma. These changes have been elucidated in rat models of experimental burn injury where the liver was isolated and perfused ex vivo. Because these studies were performed in fasted animals to deplete glycogen stores, thus simplifying quantification of gluconeogenesis, these observations reflect the combined impact of fasting and injury on liver metabolism. Herein we asked whether the metabolic response to experimental burn injury is different in fed vs. fasted animals. Rats were subjected to a cutaneous burn covering 20% of the total body surface area, or to similar procedures without administering the burn, hence a sham-burn. Half of the animals in the burn and sham-burn groups were fasted starting on postburn day 3, and the others allowed to continue ad libitum. On postburn day 4, livers were isolated and perfused for 1 hour in physiological medium supplemented with 10% hematocrit red blood cells. The uptake/release rates of major carbon and nitrogen sources, oxygen, and carbon dioxide were measured during the perfusion and the data fed into a mass balance model to estimate intracellular fluxes. The data show that in fed animals, injury increased glucose output mainly from glycogen breakdown and minimally impacted amino acid metabolism. In fasted animals, injury did not increase glucose output but increased urea production and the uptake of several amino acids, namely glutamine, arginine, glycine, and methionine. Furthermore, sham-burn animals responded to fasting by triggering gluconeogenesis from lactate; however, in burned animals the preferred gluconeogenic substrate was amino acids. Taken together, these results suggest that the fed state prevents the burn-induced increase in hepatic amino acid utilization for gluconeogenesis. The role of glycogen stores and means to increase and/or maintain internal sources of glucose to prevent increased hepatic amino acid utilization warrant further studies.

## Introduction

The liver has many complex physiological functions including detoxification, lipid, protein, and carbohydrate metabolism, as well as bile and urea production. It also plays a major role in the onset and maintenance of aberrant “hypermetabolic” patterns associated with various disease states, such as cutaneous burns, infections and major trauma, which are characterized by an accelerated breakdown of skeletal muscle protein, increased resting energy expenditure, and a negative nitrogen balance [1], [2], [3]. There is significant prior work describing the effect of experimental burn injury on liver metabolism in a rat model using the isolated perfused liver approach [4], [5], [6], [7], [8], [9], as well as in rats and rabbits using stable labeling approaches in whole animals [10], [11]. These studies showed that the hepatic response to burn injury is generally characterized by an up-regulation of glucose, fatty acid and amino acid turnover as well as increased gene expression levels of enzymes involved in the urea cycle, gluconeogenesis, and the metabolism of several amino acids.

A major goal of these studies was to determine the effect of systemic injury on gluconeogenesis; therefore, in order to eliminate the confounding effect of glycogen breakdown to the observed hepatic glucose output, animals were fasted overnight to deplete glycogen stores prior to perfusion analysis, thus simplifying measurement of de novo gluconeogenesis. Furthermore, the data were fitted to a mass balance model to estimate internal fluxes through the hepatic metabolic network where it was assumed that all strictly glycolytic reactions were inhibited thus reducing the number of unknowns as well as eliminating potential futile metabolic cycles that would be difficult to resolve [5], [6], [8], [9]. Clearly, a limitation of these studies is that the metabolic responses to burn injury involve many of the same pathways that are affected by fasting – in fact most of the pathways involved in central carbon and nitrogen metabolism – therefore the reported effect of burn injury could have been altered by the fasting response. Since burn victims are not fasted, and in fact the nutritional regimen is very important to their recovery [12], [13], it is important to better understand the effect of fasting and lack thereof on the hepatic response to burn injury. Therefore, the objective of this study is to determine the effect of fasting on the hepatic response to burn injury, and as a corollary, the effect of burn injury on the fasting response of the liver.

For this purpose, we used a standard rat burn injury model and isolated liver perfusion system that we have previously used [8], [14]. In addition, we used an extended mass balance model to include reactions that are active in a fed state (e.g. glycolysis, lipid synthesis, and glycogen synthesis), thermodynamic constraints to reduce the solution space, and an objective function-based optimization to reach a unique solution [15]. The results show that in fed animals, burn injury increased glucose and urea production but minimally impacted amino acids. On the other hand, in fasted animals, injury did not increase glucose output, further enhanced urea production, and increased amino acid utilization. The data also show that in normal rats, lactate is the main substrate used for fasting-induced gluconeogenesis; however, in burned rats, amino acids are preferentially used.

## Materials and Methods

### 1. Animal Groups

Male Sprague-Dawley rats (Charles River Labs, Wilmington, MA) weighing between 150 and 200 g were used. The animals were housed in a temperature-controlled environment (25°C) with a 12-hour light-dark cycle and provided water and standard chow ad libitum. All experimental procedures were carried out in accordance with National Research Council guidelines and approved by the Rutgers University Animal Care and Facilities Committee. The rats were first randomized into two groups of equal size. The first group received scald burn injury (described further below) and the second group received a sham burn injury. Three days post injury, food was removed from half of the animals in each group to initiate fasting. One day later (four days post injury), animals underwent isolated liver perfusion (also described further below). Thus, totally four groups were investigated: *Sham+Fed* (n = 4), *Sham+Fasted* (n = 3), *Burn+Fed* (n = 4), *Burn+Fasted* (n = 4). A total of 15 rats were used in this study.

### 2. Burn Injury

A full-thickness burn on the dorsal skin corresponding to 20% of the total body surface area (TBSA) was performed as described previously [5], [8], [16] and based on earlier studies [17], [18]. This model was chosen because it has nearly 100% long-term survival, no evidence of systemic hypoperfusion, and no significant effect on the feeding pattern [18]. Briefly, rats were anesthetized by intraperitoneal injection of 80 to 100 mg/kg ketamine +12 to 10 mg/kg xylazine. All hair was removed from the dorsal abdominal area using electric clippers. The animal’s back was immersed in water at 100°C for 10 s to produce a full-thickness scald injury over 20% TBSA. Immediately after burns, the animals were resuscitated with 50 mL/kg of saline injected intraperitoneally. Sham burn controls consisted of animals treated identically but immersed in lukewarm water. Rats were single caged after burn or sham burn and given standard rat chow and water ad libitum. No post-burn analgesics were administered, consistent with other studies since the nerve endings in the skin in full thickness burn model are destroyed and the skin becomes insensate [17]. Furthermore, after the animals woke up, they ate, drank, moved freely about the cage, responded to external stimuli, and did not show clinical signs of pain or distress. Rats were single caged after burn and standard bedding was used. Animals were checked every 12 h for signs of pain or distress (trembling, ruffled fur, hunched posture, inability to ambulate, lethargy or lack of adequate response when attempts are made to pick up the animal) until time of sacrifice (by ex vivo perfusion as described below), which was 4 days postburn. None of the animals studied here exhibited such signs and therefore all animals were sacrificed 4 days postburn as planned. All burn and sham-burn procedures were carried out in the 8 am –10 am time window period to insure that all animals were at the same stage in their circadian cycle [19], [20], [21].

### 3. Liver Perfusion

Three days post injury, food was removed from half of the animals in the sham burn and burn groups to initiate fasting. One day later (four days post injury), animals underwent isolated liver perfusion following a modification of Mortimore’s methods [22], and described in greater detail in a recent publication [14]. Briefly, the perfusion system consisted of a medium reservoir, heat exchanger, oxygenator, bubble trap, and liver perfusion chamber in a closed loop. Perfusate medium recirculation by a peristaltic pump was initiated and maintained to stabilize the system prior to rat liver preparation. The rats were anesthetized with an intraperitoneal injection of ketamine (80 to 100 mg/kg) and xylazine (12 to 10 mg/kg). The abdominal cavity was opened and heparin (1000 U/kg) was injected by transphrenic cardiac puncture, and then the liver was cannulated *in situ* via the portal vein and immediately perfused with perfusate solution. The hepatic artery and the suprarenal vena cava were ligated, and the liver outflow from the hepatic vein collected through a catheter inserted into the inferior vena cava via the right atrium. After blood was washed out from the liver by flushing with perfusate for 10 minutes, the end of the tube connecting to the outflow catheter was placed into the perfusate reservoir to initiate the recirculating perfusion. Death of the animal (as evidenced by irreversible respiratory arrest) occurred in the first few seconds of perfusion due to exsanguination. The perfusate composition is provided in Table S1, with pH adjusted to 7.4, supplemented with washed bovine red blood cells (RBCs) (Lampire, Pipersville, PA) at 10% hematocrit and oxygenated by passing through 3 m of silicone tubing in contact with a 95% O_2_/5% CO_2_ gas mixture [14]. The perfusion pressure was kept below 15cm H_2_O and the flow rate was set to 3.0 ml/min/g liver. Total initial perfusate volume was 500 ml. The temperature of the heating fluid in the heat exchanger was set to 50°C to account for incomplete heat transfer and heat losses between the heat exchanger and the liver inlet in order to maintain the liver at physiological temperature (37.0±0.5°C), as measured by inserting a thermocouple between the lobes.

Detailed explanations regarding the measurements of metabolite concentrations in the perfusate can be found elsewhere [9], [14], [23]. Briefly, the dissolved O_2_ and total hemoglobin concentrations were immediately measured using a blood gas analyzer (Bayer Rapidlab 855, Diamond Diagnostics, MA). Several metabolites were measured using commercial assay kits (urea and β-hydroxybutyrate: Stanbio Laboratory, Boerne, TX; glucose and glutamine: Sigma Chemical; lactate: Trinity Biotech, Berkeley Heights, NJ). Individual amino acid concentrations in the perfusate samples were quantified by an automated high-performance liquid chromatography (HPLC) system which consisted of a Waters 2690 Separations Module and Waters 474 Scanning Fluorescence Detector (Waters Co., Milford, MA) set at an excitation of 250 nm and an emission of 395 nm.

Cell lysis was assessed based on the release of intracellular lactate dehydrogenase (LDH) into the perfusate. LDH activity measurements from perfusate samples were carried out using a cytotoxicity detection kit (Roche, Indianapolis, IN). To estimate the number of cells lysed based on measured LDH activity, values were compared to LDH activity measured from a known number of freshly isolated rat hepatocytes. Note that in separate control experiments where perfusions were carried out without livers in the circuit, no significant changes in metabolite levels in the perfusate were noted (data not shown), suggesting that the contribution of RBCs to the metabolite concentration changes observed with the livers were negligible.

Multiple comparisons among the groups were performed using analysis of variance (ANOVA) followed by Tukey’s studentized range test. The criterion for statistical significance used was P<0.05.

### 4. Metabolic Network Analysis

#### Metabolic network

The liver metabolic network used in this work is based on networks originally developed for perfused livers and hepatocyte cultures [5], [8], [24], and was modified to simultaneously include both glycolytic and gluconeogenic pathways, fatty acid synthesis and oxidation, as well as glycogenesis and glycogenolysis [15]. This hepatic network model is described extensively in reference [15], and the major points are recapitulated below. Given the physiological properties of the liver and the perfusate composition, the network involves all major liver-specific pathways involved in central carbon and nitrogen metabolism such as gluconeogenesis, glycolysis, urea cycle, fatty acid metabolism, pentose phosphate pathway, TCA cycle, glycogen metabolism and amino acid metabolism (see Table 1). The major assumptions in the network model are similar to that used in prior studies [5], [8], [24], [25], [26], [27] and can be summarized as follows:

**Table 1:** Hepatic Network Model* #.

Reaction no	Enzymes and explanations	Glycolysis & Gluconeogenesis	Sham+Fed	Sham+Fasted	Burn+Fed	Burn+Fasted
Reaction 1	Glucose-6-Pase	Glucose-6-P+H2O = = >Glucose+Pi	**111.43±14.34**	**68.90±19.43**	**164.07±22.70**	**57.87±11.24**
Reaction 2	Phosphoglucose isomerase	Fructose-6-P< = = > Glucose-6-P	−42.79±81.57	−12.90±89.47	−34.05±76.36	−2.64±62.31
Reaction 3	Fructose-1,6-Pase-1	Fructose-1,6-P2+ H2O = = >Fructose-6-P+Pi	19.39±19.39	38.28±38.28	21.16±21.16	29.83±29.83
Reaction 4	Triose P-isomerase, fructose biphosphate aldolase	2 Glyceraldehyde-3-P< = = > Fructose-1,6-P2	−42.79±81.57	−12.90±89.47	−34.05±76.36	−2.64±62.31
Reaction 5	Glyceraldehyde-P dehydrogenase, 3-phosphoglycerate kinase, phosphoglyceromutase, enolase	ATP+NADH+PEP+H++H2O< = = >Glyceraldehyde-3-P+Pi+NAD++ADP	−94.09±157.32	−29.86±166.70	−78.43±146.99	−16.07±120.05
Reaction 6	PEPCK	Oxaloacetate+GTP< = = > CO2+ PEP+GDP	32.93±30.30	68.42±68.42	35.91±32.65	74.30±29.68
Reaction 7	Pyruvate carboxylase	CO2+ ATP+Pyruvate+H2O = = >Pi+ADP+Oxaloacetate	29.53±29.53	71.00±71.00	32.65±32.65	29.68±29.68
Reaction 8	Hexokinase	Glucose+ATP = = >Glucose-6-P+ADP	134.94±134.94	107.95±107.95	122.86±122.86	91.96±91.96
Reaction 9	PFK-1	Fructose-6-P+ATP = = >Fructose-1,6-P2+ ADP	62.18±62.18	51.18±51.18	55.21±55.21	32.48±32.48
Reaction 10	Pyruvate kinase	ADP+PEP = = >ATP+Pyruvate	0.00±0.00	0.00±0.00	0.00±0.00	0.00±0.00
		**PPP**				
Reaction 11	Glucose-6-P dehydrogenase and 3 additional steps	Glucose-6-P +12 NADP+ +7 H2O = = >6 CO2+12 NADPH+Pi +12 H+	19.10±19.10	40.80±40.80	41.14±41.14	34.19±34.19
		**Lactate metabolism**				
Reaction 12	Lactate dehydrogenase	NAD++Lactate< = = >NADH+Pyruvate+H+	**13.03±21.03**	**105.76±18.76**	**17.15±21.27**	**6.31±15.23**
		**TCA cycle**				
Reaction 13	PDH	NAD++Pyruvate+CoA-SH = = >CO2+ NADH+Acetyl-CoA+H+	125.89±125.89	146.68±146.68	116.24±116.24	86.45±86.45
Reaction 14	Citrate synthase	Oxaloacetate+Acetyl-CoA+H2O = = >Citrate+CoA-SH+H+	63.13±50.50	72.00±67.05	73.85±73.85	62.86±62.86
Reaction 15	Aconitase, isocitrate dehydrogenase	NAD++Citrate< = = >CO2+ NADH+a-ketoglutarate	63.13±50.50	72.00±67.05	73.85±73.85	62.86±62.86
Reaction 16	a-ketoglutarate dehydrogenase	NAD++CoA-SH+a-ketoglutarate = = >CO2+ NADH+Succinyl-CoA+H+	59.25±57.35	73.77±71.66	79.45±76.91	109.80±67.89
Reaction 17	Succinyl-CoA synthase and succinate dehydrogenase	Pi+GDP+Succinyl-CoA+FAD< = = >GTP+CoA-SH+Fumarate+FADH2	71.51±69.10	73.06±73.06	80.65±77.21	111.40±68.01
Reaction 18	Fumarase	Fumarate+H2O< = = >Malate	88.48±71.23	88.01±71.99	104.12±78.64	128.69±75.67
Reaction 19	Malate dehydrogenase	NAD++Malate< = = >NADH+Oxaloacetate+H+	88.48±71.23	88.01±71.99	104.12±78.64	128.69±75.67
		**Urea metabolism**				
Reaction 20	Arginase	Arginine+H2O = = >Urea+Ornithine	**17.08±2.05**	**11.62±2.60**	**29.04±1.43**	**35.68±7.28**
Reaction 21	Carbonate dehydratase, carbamoyl-P synthase, ornithine transcarbamylase	CO2+2 ATP+Ornithine+NH4++H2O < = = >2 Pi +2 ADP+Citrulline +3 H+	13.52±4.63	6.94±6.94	18.82±4.91	11.42±10.46
Reaction 22	Argininosuccinate synthetase, argininosuccinase.	ATP+Citrulline+Aspartate = = >Fumarate+Arginine+AMP+PPi	13.52±4.63	6.94±6.94	18.82±4.91	11.42±10.46
		**Amino acid metabolism**				
Reaction 23	Alanine aminotranferase	NAD++Alanine+H2O< = = >NADH+Pyruvate+NH4++H+	**2.18±1.24**	−**2.04±2.30**	**2.67±2.30**	**2.80±0.96**
Reaction 24	Serine dehydratase	Serine = = >Pyruvate+NH4+	12.09±12.09	11.60±11.60	11.85±11.85	12.94±12.94
Reaction 25	Transaminase, 3-mercaptopyruvate sulfurtransferase	NAD++Cysteine+H2SO3+ H2O< = = >NADH+Pyruvate+NH4++H2S2O3+ H+	0.82±20.31	0.45±20.45	0.99±20.08	1.83±20.35
Reaction 26	Threonine 3-dehydrogenase, acetyl-CoA ligase	NAD++CoA-SH+Threonine = = >NADH+Acetyl-CoA+Glycine	**2.65±2.65**	**1.13±1.13**	**1.05±1.05**	**4.78±0.57**
Reaction 27	Glycine hydroxymethyltranferase, glycine cleavage system	NAD+ +2 Glycine< = = >CO2+ NADH+NH4++Serine	**2.65±2.04**	**1.32±1.87**	**3.73±0.89**	**6.39±0.98**
Reaction 28	Lysine metabolism (8 steps)	5 NAD++CoA-SH+FAD+Lysine +3 H2O = = >2 CO2+5 NADH+FADH2+2 NH4++Acetoacetyl-CoA +5 H+	0.92±0.92	0.27±0.27	10.33±10.33	1.33±1.33
Reaction 29	Phenylalanine hydroxylase	Phenylalanine+Tetrahydrobiopterin+O2 = = > Dihydrobiopterin+Tyrosine+H2O	2.68±1.19	1.93±1.93	3.31±0.74	3.20±0.23
Reaction 30	Tyrosine metabolism (5 steps)	NAD+ +2 O2+ Tyrosine+H2O = = >CO2+ NADH+Fumarate+NH4++H++Acetoacetate	2.82±1.86	10.22±10.22	2.25±1.08	3.08±0.76
Reaction 31	Glutamate dehydrogenase, aminotransferase	NAD++Glutamate+H2O+ NADP+< = = >NADH+a-ketoglutarate+NH4+ NADPH+H+	**13.63±11.73**	**9.73±14.28**	**13.62±11.08**	**56.17±14.27**
Reaction 32	Glutaminase	Glutamine+H2O = = >NH4++Glutamate	**12.90±5.93**	**17.82±5.89**	**11.15±3.94**	**44.82±4.04**
Reaction 33	Ornithine metabolism (2 steps)	NADP++NAD++Ornithine+H2O = = >NADPH+NADH+NH4++Glutamate+H+	**2.83±2.83**	**4.83±4.83**	**8.52±4.35**	**18.45±7.64**
Reaction 34	Proline oxidase, 1-pyrroline-5-carboxylate dehydrogenase	0.5 NADP+ +0.5 NAD+ +0.5 O2+ Proline = = >0.5 NADPH +0.5 NADH+Glutamate+H+	1.74±0.73	2.15±2.15	2.54±2.01	4.17±0.52
Reaction 35	Histidine metabolism (4 steps)	Histidine+THF +2 H2O = = >NH4++Glutamate +2-formimino-THF	**2.74±1.14**	**1.71±1.71**	**3.71±1.19**	**5.20±0.88**
Reaction 36	Methionine metabolism (5 steps)	ATP+NAD++CoA-SH+Serine+Methionine = = >CO2+ Pi+NADH+NH4++Cysteine+PPi+Adenosine+Propinoyl-CoA	**0.82±0.31**	**0.60±0.60**	**0.99±0.08**	**1.83±0.35**
Reaction 37	Propinoyl-CoA carboxylase, Methylmalonyl-CoA epimerase, Methylmalonyl-CoA mutase	CO2+ ATP+Propinoyl-CoA = = >Succinyl-CoA+AMP+PPi	1.79±1.28	1.74±1.74	2.07±1.16	2.34±0.85
Reaction 38	Aspartate aminotransferase	NAD++Aspartate+H2O< = = >NADH+Oxaloacetate+NH4++H+	−7.39±11.49	−4.51±9.97	−12.88±12.20	−9.09±13.89
Reaction 39	Asparaginase	Asparagine+H2O = = >NH4++Aspartate	10.00±10.00	9.82±9.82	10.50±9.00	10.00±9.50
Reaction 40	Valine metabolism (7 steps)	0.5 NADP+ +3.5 NAD++FAD +2 H2O+valine = = >2 CO2+0.5 NADPH +3.5 NADH+FADH2+ NH4++Propinoyl-CoA +3 H+	0.93±0.93	0.30±0.30	0.34±0.34	0.43±0.43
Reaction 41	Isoleucine Metabolism (6 steps)	0.5 NADP+ +2.5 NAD++FAD +2 H2O+isoleucine = = >CO2+0.5 NADPH +2.5 NADH+Acetyl-CoA+FADH2+ NH4++Propinoyl-CoA +3 H+	0.05±0.05	0.84±0.84	0.74±0.74	0.08±0.08
Reaction 42	Leucine Metabolism (6 steps)	0.5 NADP++ATP +1.5 NAD++FAD+H2O+leucine = = >0.5 NADPH+Pi+ADP +1.5 NADH+Acetyl-CoA+FADH2+ NH4+ +2 H++Acetoacetate	0.86±0.86	0.06±0.06	1.13±1.13	0.13±0.13
		**Lipid metabolism**				
Reaction 43	Hepatic Lipase, Glycerol-3-P dehydrogenase	Palmitoylglycerol+NAD+ +3 H2O< = = >Glyceraldehyde-3-P +3 Palmitate+NADH +4 H+	3.80±10.94	2.22±14.07	6.31±9.76	8.44±6.92
Reaction 44	Fatty acid oxidation (7x4 steps)	ATP +7 NAD++Palmitate +8 CoA-SH +7 FAD = = >2 Pi +7 NADH +8 Acetyl-CoA +7 FADH2+ AMP	21.84±21.84	24.44±24.44	24.10±24.10	25.33±20.75
Reaction 45	Fatty acid synthesis (7x4 steps)	14 NADPH +7 ATP +8 Acetyl-CoA +14 H+ = = >14 NADP+ +7 Pi+Palmitate +7 ADP +6 H2O	10.71±10.71	17.78±17.78	5.16±5.16	0.00±0.00
Reaction 46	Thiolase (Ketogenesis)	2 Acetyl-CoA < = = >2 CoA-SH+Acetoacetyl-CoA	79.97±81.80	91.03±91.57	75.89±96.55	84.75±87.41
Reaction 47	HMG-CoA synthase and lyase (Ketogenesis)	Acetoacetyl-CoA+H2O = = >CoA-SH+Acetoacetate	80.89±80.89	91.30±91.30	86.22±86.22	86.08±86.08
Reaction 48	B-OH-butyrate dehydrogenase (Ketogenesis)	NADH+H++Acetoacetate< = = >NAD++B-OH-butyrate	**59.20±3.53**	**74.68±7.93**	**69.38±4.24**	**71.24±3.24**
		**Glycogen metabolism**				
Reaction 49	Glycogenesis	Glucose-6-P+ATP+H2O+Glycogen(n-1) = = >ADP+Glycogen	0.00±0.00	13.06±13.06	0.00±0.00	6.52±6.52
Reaction 50	Glycogenolysis	Pi+Glycogen = = >Glucose-6-P+Glycogen(n-1)	178.34±112.69	84.01±84.01	220.55±121.49	69.46±69.46
		**Electron transport reactions**				
Reaction 51	Electron transport system	3 ADP+NADH +0.5 O2+ H+ = = >3 ATP+NAD++H2O	385.33±114.67	358.76±141.24	390.32±109.68	416.59±83.41
Reaction 52	Electron transport system	2 ADP+FADH2+0.5 O2 = = >2 ATP+FAD+H2O	202.43±138.51	182.22±182.22	243.79±138.65	286.19±109.07

* Mean values of fluxes given in bold are significantly different (ANOVA, P<0.05; N = 3) from each other.

# While reactions 1–7 are gluconeogenic, reaction 2 (generation of glucose-6-P from fructose-6-P), and reactions 4–5 (generation of phosphoenolpyruvate [PEP] from glyceraldehyde-3-P and frucrose-1,6-P2) are also utilized by the glycolysis pathway in a reverse direction. Reaction 1 (catalyzed by glucose-6-phosphatase) is strictly gluconeogenic, and is opposed by glycolytic reaction 10 (glucokinase). Gluconeogenic reaction 3 (fructose-1,6-bisphosphatase) and glycolytic reaction 9 (phosphofructokinase) also take place in opposite directions.

- The model considers the major routes of carbon and nitrogen metabolism, more specifically glycolysis/gluconeogenesis, glycogenolysis/glycogenesis, fatty acid synthesis/oxidation, tricarboxylic acid cycle, urea cycle, major transamination and deamination reactions involved in anaplerosis;
- Phase I- and phase II- dependent detoxification reactions are not included because they do not significantly impact on the overall carbon and nitrogen balances;
- The pentose phosphate pathway (PPP) considers the oxidative branch as the nonoxidative branch is primarily used for nucleotide synthesis, which is not significant in a nonproliferating liver;
- Many of the reactions involved in the de novo biosynthesis of amino acids are ignored because the fluxes involved are much smaller than those in carbohydrate and fat metabolism;
- Protein synthesis and breakdown were also not considered in the model because they accounts for small portion of the nitrogen metabolism in the perfused livers;
- The model does not attempt to close an energy balance around ATP because much of the ATP consumption is by ionic transport processes that are difficult to quantify;
- Pseudo steady-state is assumed for the duration of perfusion (except for the first few minutes), which is a reasonable assumption because concentrations of metabolites in the perfusate reservoir changed linearly, implying that the perfused liver was metabolically stable for the duration of the perfusion;
- When two metabolic pathways in opposite directions form a futile cycle, it is assumed that only one of the two directions is active and the other is inactive (in other words the forward and reverse pathways cannot be active at the same time). In general, reciprocal control between the enzymes involved in such pathways exists to minimize the formation of futile cycles, and this constraint was applied using a mixed integer binary formulation as described further below.

#### Intracellular flux ranges

Mass balances were performed around 50 intracellular metabolites (full list in Table S4), thus applying the same number of mass balance constraints. Note that cofactors including ATP and NADPH were not balanced because several reactions using these were not quantified. However, NADH was balanced because most of the reactions involved with this metabolite are included in the model [5], [6], [8], [9], [28]. In addition, we included thermodynamic constraints whereby pathway direction must be thermodynamically feasible and an exergonic reaction can be a “driving-force” for an endergonic reaction if these two reactions are coupled in the same pathway, as described in previous reports [15], [26], and also summarized below.

**Figure 1:**
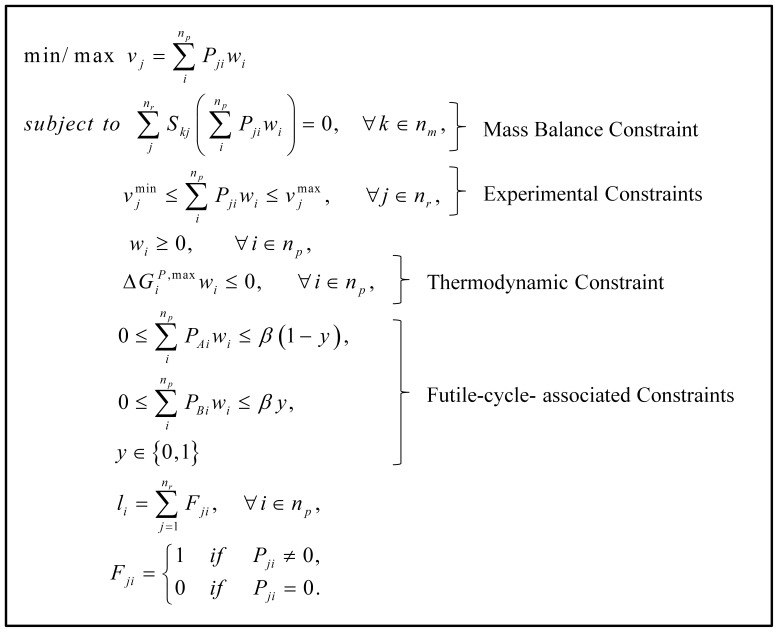
Mathematical model utilized to calculate the flux ranges. See text for detailed explanations.

The mathematical formulation used in this study to calculate the intracellular fluxes has been already described in great detail in our previous study [15]. Given the fact that every flux vector ***v*** in a metabolic network can be expressed as a linear combination of elementary modes; 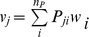 (where *w_i_* is the weight of pathway *i*; *P_ji_* corresponds to the relative flux level of reaction *j* in pathway *i*; and *n_p_* is the total number of pathways), a mixed integer linear programming (MILP) was used to calculate the intracellular fluxes as given in Figure 1. *S_kj_* is the stoichiometric coefficient of metabolite *k* in reaction *j*; *v_j_* is the flux of reaction *j*; *n_m_* and *n_r_* are the total number of metabolites, and total number of reactions, respectively. Mass balance constraints and other constraints based on experimental measurements are described by elementary modes with their corresponding weight values as shown in Figure 1. The Gibbs free energy of the pathway *i*, 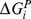, is the summation of the Gibbs free energies of reactions involved in that pathway which should be less than or equal to zero. The condition implies that the pathway *i* can be active (*w_i_≥0*) if its Gibbs free energy is less than zero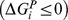, otherwise it is not active (*w_i_ = 0*). Using the maximum and minimum values of concentrations of hepatic metabolites [29], we can identify a range of values for the Gibbs free energy of each pathway, 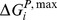
and 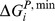. Since *w_i_* is a positive variable, any *w_i_* satisfying the inequality 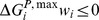 also satisfies 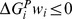 and 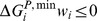. Thus, the constraint described by 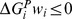 which is nonlinear is replaced by 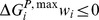 that further tightens the solution space. The indices *A* and *B* represent any two reactions forming a futile cycle. This constraint describes the reciprocal control between two reactions (such as the reaction of glucokinase and the reaction of glucose-6-phosphatase) forming a futile cycle. It allows the two reactions (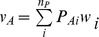
and 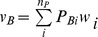) to inhibit each other and prevent the formation of the cycle. *y* is a binary variable, and β is a very large number which forces *y* to be 0 (thus *v_B_* is zero) if the reaction *v_A_* is active. If the reaction *v_A_* is zero, then *v_B_* can take any value.

The minimum and maximum experimental values of measured fluxes used in the model (Figure 1) are listed in Table S3. Intracellular flux ranges were calculated by minimizing or maximizing each intracellular reaction rate shown in Figure 1. This formulation provides the maximum and minimum values that a flux can take: 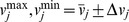 (where 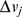 corresponds to error, and 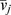 is the mean value of 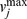 and 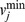). Comparisons among fluxes of the four groups were performed using analysis of variance (ANOVA) followed by Tukey’s studentized range test considering the minimum, maximum and mean values of each flux which were calculated. The criterion for statistical significance was chosen as P<0.05. For the optimization routine, the upper and lower limits of each unknown flux was assumed to be ±500 µmol/g liver/h based on a survey of a large set of prior perfused liver studies in burned rats [5], [6], [8], [9]. The elementary modes were calculated using a MATLAB package, *CellNetAnalyzer* [30], and the mixed integer linear programming problem was solved using GAMS/CPLEX.

#### Determining unique solutions for intracellular fluxes

Because the above framework leads to an underdetermined system, in order to obtain a single solution for each condition, we applied an optimization criterion which states that the activity of short pathways is maximized [15], and which is based on a prior analysis of liver perfusion studies showing that short pathways tend to involve many liver-specific pathways which have higher weights [31]. The objective function of this problem was stated as:
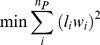
(1)which forces shorter pathways to take larger weight values. The length (*l)* of any pathway is equal to the number of reactions involved in that pathway and is calculated using the binary matrix of elementary modes, *F*, where *F_ji_* is equal to 1 if *P_ji_* is different than zero, otherwise *F_ji_* is zero (Figure 1). The thermodynamic, experimental, and futile cycle-related constraints were also applied as shown in Figure 1. This assumption is consistent with other studies suggesting that the activity of short pathways is relatively high and that these pathways tend to be organism-specific [32], [33]. Solving this problem involves mixed integer quadratic programming (MIQP), which was done using GAMS/CPLEX.

## Results

### 1. Extracellular Fluxes

We investigated the metabolic response of liver to fasting and/or burn systemic injury in rats. Totally, four different animal groups, *Sham+Fed*, *Sham+Fasted*, *Burn+Fed*, *Burn+Fasted*, were compared. The average uptake and release rates of major metabolites, including glucose, urea, lactate, β-hydroxybutyrate, oxygen, and amino acids were measured from perfused livers isolated 4 days post injury. For fasted groups, fasting was applied 24 h prior to perfusion. The complete set of data is provided in Tables S2 and S3. Below we focus on the most interesting individual metabolite findings and results from system-wide metabolic flux analyses.

**Figure 2:**
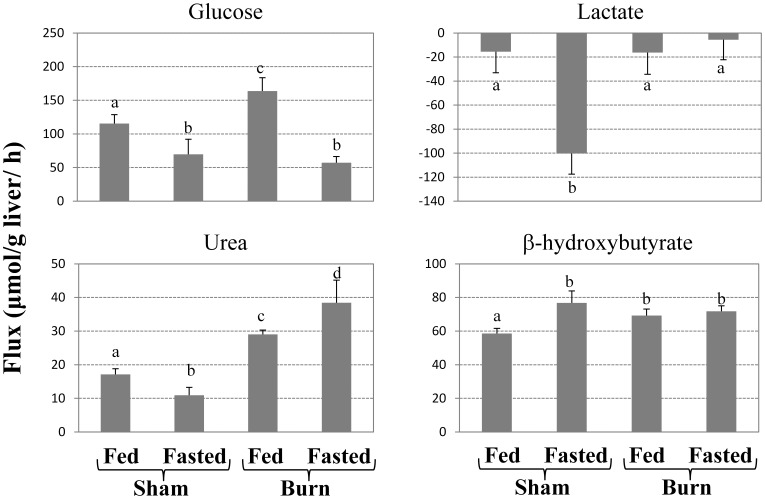
Average glucose, lactate, urea and β-hydroxybutyrate fluxes in perfused livers. Bars above and below indicate net production and uptake, respectively. Data shown were obtained from the slope of linear fits of measured perfusate concentrations vs. time normalized to liver mass. Bars labeled with different letters are statistically significantly different (ANOVA, P<0.05; N≥3) from each other. For example, lactate secretion rates in *Sham+Fed* group (a), *Burn+Fed* group (a) and *Burn+Fasted* group (a) are not significantly different from each other, whereas *Sham+Fasted* (b) is significantly different from the other groups. Data shown are means±SD.

Figure 2 shows glucose, lactate, urea and β-hydroxybutyrate production or utilization rates in the four different groups. Following fasting, glucose production rates were significantly decreased in both the burn and sham-burn groups. Interestingly, glucose production increased as a result of burn injury in the fed groups (*Burn+Fed* vs. *Sham+Fed*), while no significant effect, and even a trend in the opposite direction (i.e. glucose production decreased as a result of burn injury) was observed in the fasted groups (*Burn+Fasted* vs. *Sham+Fasted*). The uptake of lactate, a major substrate for the gluconeogenic pathway, was increased over 5 fold by fasting in the sham groups, but not in the burned groups. As expected, burn injury significantly increased urea production when evaluated in both fed and fasted conditions. Of note is that the magnitude of the increase was less than 2 fold in the fed groups, but more than 3.5 fold in the fasted groups. In addition, fasting reduced urea production in the sham groups but increased it in the burned animals. Beta-hydroxybutyrate production was significantly – albeit slightly – increased by fasting in the sham groups, but not in the burned goups. In addition, beta-hydroxybutyrate production in the *Burn+Fed* and *Burn+Fasted* groups was at the same elevated level seen in the *Sham+Fasted* group.

**Figure 3:**
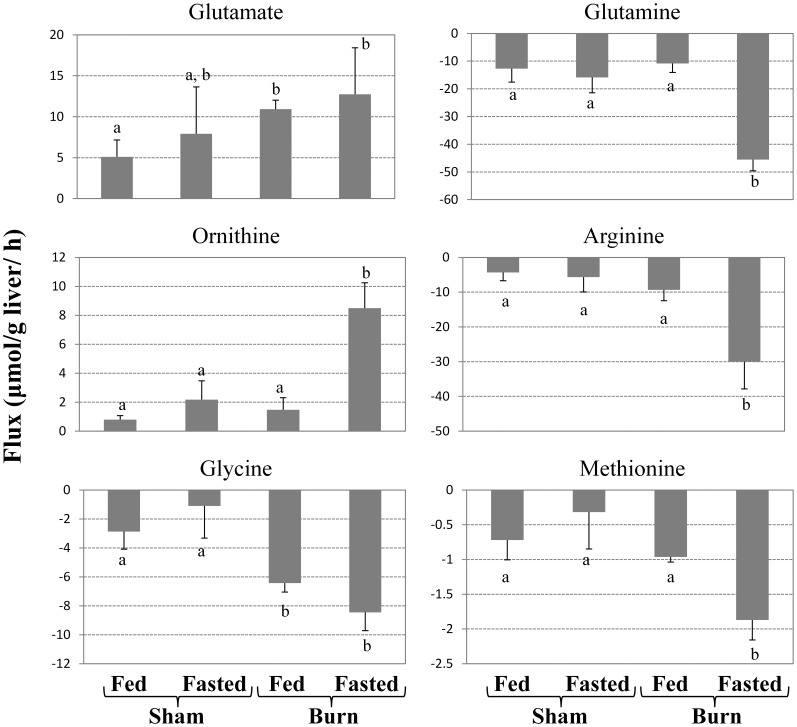
Average fluxes for the main amino acids in perfused livers. Bars above and below indicate net production and uptake, respectively. Data shown were obtained from the slope of linear fits of measured perfusate concentrations vs. time normalized to liver mass. Bars labeled with different letters are statistically significantly different (ANOVA, P<0.05; N≥3) from each other. Data shown are means±SD.

Among the amino acids, the uptake/release rates of glutamine, ornithine, arginine, glycine and methionine showed significant variations among the groups (Figure 3). Glutamate production rates were higher in the burned groups when compared to the *Sham+Fed* group; however, there was no significant effect of fasting in the sham-burned groups or in the burned groups. Glutamine, arginine, and methionine utilization rates and ornithine production rate were significantly higher in the *Burn+Fasted* group than the other groups, whereas no significant differences were observed among the other groups.

**Figure 4:**
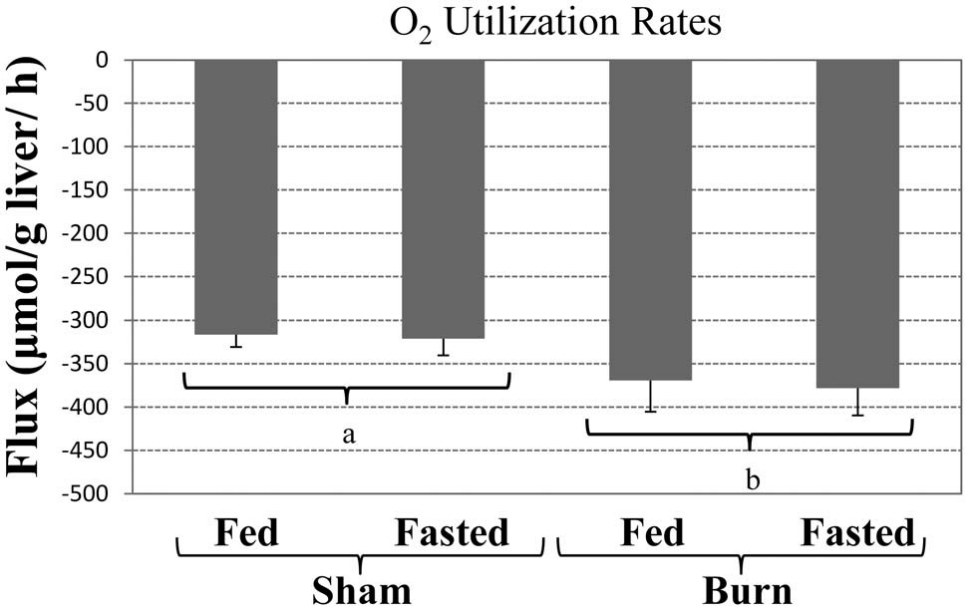
Average oxygen utilization rates in perfused livers. Data shown were obtained from the measured decrease in oxygen tension and hemoglobin saturation between the outlet and inlet of each perfused liver. Bars labeled with different letters are statistically significantly different (ANOVA, P<0.05; N≥3) from each other. Data shown are means±SD.

Oxygen consumption rates were slightly (about 15–20%) but significantly elevated in response to burn injury when comparing all the burn groups to all the sham groups (Figure 4). Several other metabolites were measured as well, but their rates were not significantly affected by burn and/or fasting; the specific values are provided in Table S2.

**Figure 5:**
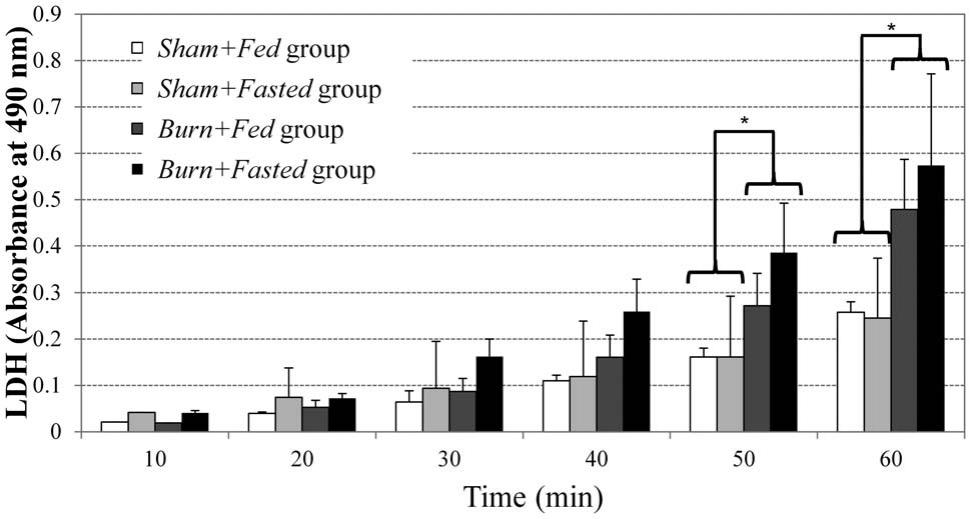
Accumulation of lactate dehydrogenase activity in perfusate as a function of time during the perfusion. N≥3 for each group. * indicates significantly higher activity in the burned groups compared to the sham-burned groups (ANOVA, P<0.05). Data shown are means±SD.

To assess tissue damage, we monitored the release of intracellular lactate dehydrogenase (LDH) during the perfusion (Figure 5). All groups showed an increase in LDH activity as a function of time. By the end of the 1 h perfusion, LDH accumulation was approximately twice in the burn groups compared to the sham groups. These results indicate that prior burn injury results in greater tissue damage during perfusion. Note that based on absorbance values obtained from lysed hepatocytes isolated from normal rat livers, we can estimate the extent of damage to about 10% of the liver mass in the burned groups at 60 min, and 5% in the sham-burned groups. These values were deemed too small to take into account in the flux model.

### 2. Intracellular Fluxes

#### Steady state flux ranges

The uncertainty of each intracellular flux was determined by calculating the minimum and maximum values that each intracellular flux can take by using experimentally determined extracellular fluxes, thermodynamic and futile cycle constraints (see Materials and Methods and Figure 1). Mean values of fluxes with their ranges are given in Table 1. Bold flux values indicate that mean values of the four different groups were found to be significantly different from each other (using ANOVA, P<0.05).

Consistent with experimental results (Figure 2), the rate of generation of glucose from glucose-6-P (reaction 1) was generally higher in the fed groups compared to the fasted groups, and was highest in the *Burn+Fasted* group (Table 1). Glucose production from glucose-6-P is dependent upon fluxes through the gluconeogenic reactions (reactions 1–6) as well as glycogen breakdown (reaction 50). However, we cannot ascribe the observed differences to a particular reaction due to the significant overlap in the flux ranges among the groups for the gluconeogenic and glycogenolytic fluxes. The uncertainty at this particular branch point reflects the lack of a direct measurement of glycogen breakdown rate. A different methodology is used further below to uniquely determine the flux distributions.

The influx of lactate and pyruvate into oxaloacetate (reaction 12) was significantly upregulated in the *Sham+Fasted* group (Table 1). Interestingly, the glycolytic reaction where pyruvate is produced from PEP by pyruvate kinase (reaction 10) was found to be inactive in all groups (Table 1). Consistent with experimental observations summarized in Figures 2 and 3 as well as Table 1, intracellular fluxes related to urea production (reaction 20), amino acid metabolism including alanine (reaction 23), glycine (reactions 26 and 27), glutamine & glutamate (reactions 31–34), histidine (reaction 35) and methionine (reaction 36), as well as β-hydroxybutyrate production (reaction 48), were found to be statistically significantly different among the groups and significantly up-regulated in the *Burn+Fasted* group.

#### Unique solutions for intracellular fluxes

**Figure 6:**
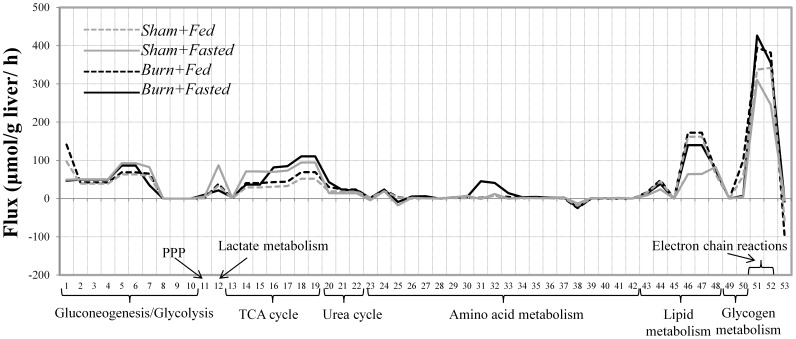
Calculated internal fluxes of perfused livers. An objective function maximizing the activity of shorter pathways was used to calculate the fluxes.

**Figure 7:**
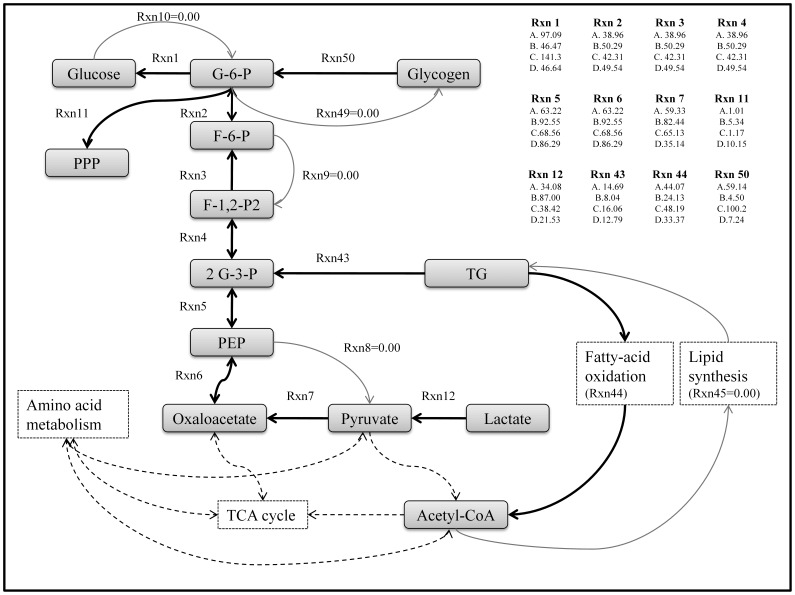
Detailed flux distribution map in the glycolysis, gluconeogenesis and lipid metabolism pathways. Note that several reaction pairs (Rxn 1 vs. Rxn 10; Rxn 3 vs. Rxn 9; Rxn 6 vs. Rxn 8; Rxn 49 vs. Rxn 50; and Rxn 44 vs. Rxn 45) form futile cycles. Thick black lines indicate active (or dominant) reactions whereas gray lines represent inactive (i.e. flux = 0) reactions. Dashed lines depict reactions not shown in detail. Positive flux values for reversible reactions which are in the gluconeogenic pathway in the figure indicate that gluconeogenesis is active. Fluxes of irreversible reactions should be equal or greater than zero. Directions of reactions are given in Table 1. Flux units are µmol/g liver/h. A: Sham+Fed group; B: Sham+Fasted group; C: Burn+Fed group; D: Burn+Fasted group.

**Figure 8:**
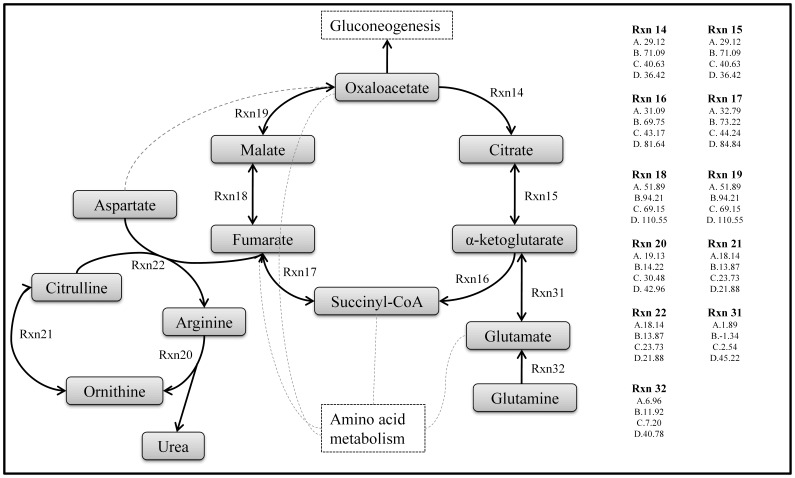
Detailed flux distribution map in the TCA and urea cycles. Thick black lines indicate active (or dominant) reactions. Dashed lines depict reactions not shown in detail. Positive flux values of reversible reactions indicate a net oxaloacetate production from citrate (for TCA cycle reactions), arginine and urea production from ornithine and citrulline (for urea cycle), and α-ketoglutarate production from glutamate. Fluxes of irreversible reactions should be equal or greater than zero. Directions of reactions are given in Table 1. Flux units are µmol/g liver/h. A: Sham+Fed group; B: Sham+Fasted group; C: Burn+Fed group; D: Burn+Fasted group.

Mass balance analysis alone did not yield a unique solution since the number of experimentally measured parameters was insufficient to fully determine the intracellular fluxes, and for this reason we reported flux ranges in Figure 1. To obtain a unique solution, we implemented an objective function which chooses an optimum point (a single solution) in the steady state flux cone defined by experimentally measured fluxes and thermodynamic constraints [15]. In addition, we used an objective function that maximizes the activity of short pathways [31]. This assumption is consistent with other studies suggesting that the activity of short pathways is relatively high [32], [33]. The calculated fluxes for all four groups are summarized in Figure 6 (numerical values are given in Table S5). This overall picture shows variations among groups in most aspects of metabolism, except for a few reactions in glycolysis and amino acid metabolism. Fluxes exhibiting significant differences among the groups are described in further detail in Figures 7 and 8. Figure 7 shows a detailed picture of fluxes in the glycolytic and gluconeogenic pathways. Applying the model to the experimental data, we found that glycolytic reactions (reactions 8, 9, and 10), glycogenesis (reaction 49), and lipid synthesis (reaction 45) were inactive, thus eliminating all possible futile cycles in the network.

The rate of generation of glucose from glucose-6-P (reaction 1) was approximately 3 times the rate of generation of glucose-6-P from fructose-6-P (reaction 2) in the fed groups (groups A&C), and the difference was mainly due to the contribution of glycogen breakdown (reaction 50). Fasting decreased the glucose production (reaction 1) flux by 2–3 fold in the sham and burn groups (groups B&D vs. A&C), and this was largely due to a concomitant >10 fold decrease in glycogen breakdown (reaction 50). In the fasted groups, glucose-6-phosphatase (reaction 1) fluxes were approximately 10% less than phosphoglucose isomerase (reaction 2) fluxes due to increased removal of glucose 6-P towards the PPP (reaction 11) in fasted animals compared to fed animals. Fasting slightly increased internal gluconeogenic fluxes (reactions 2, 3, 4, 5, 6) in both the burn (group D vs. C) and the sham (group B vs. A) groups, while the burn effect on these fluxes was minimal.

The influx of lactate and pyruvate into oxaloacetate (reactions 7 and 12), a well-known gluconeogenic route, were upregulated by fasting in the sham groups (group B vs. A) while the opposite was observed in the burn groups (group D vs. C). These findings parallel the fasting-induced changes in lactate uptake previously shown in Figure 2. In the sham groups, fasting increased the flux from oxaloacetate to PEP (reaction 6) as well as the supply of oxaloacetate from pyruvate (reaction 7); in the burn groups, fasting also increased the flux from oxaloacetate to PEP (reaction 6) but decreased the supply of oxaloacetate from pyruvate (reaction 7); therefore, to maintain steady state, the supply of oxaloacetate must have come from other sources, and more specifically from the TCA cycle, as described further below.

Fatty acid oxidation (reaction 44) was found to be active by the model in all groups while the opposing fatty acid synthesis (reaction 45) was found to be inactive. Fatty acid oxidation was generally higher in the fed groups (groups A and C) than the fasted groups (groups B and D). In addition, burn injury increased fatty oxidation flux minimally in the fed groups (groups A vs. C) and more significantly in the fasted groups (groups B vs. D).

Figure 8 provides a detailed picture of the flux changes around the TCA and urea cycles. α-ketoglutarate generation from oxaloacetate (reactions 14 and 15) was increased in the *Sham+Fasted* group (group B) compared to other groups. Oxaloacetate production from α-ketoglutarate represented by reactions 16–19 fluxes was higher in both fasted groups (groups B and D) compared to the fed groups (groups A and C). Comparing α-ketoglutarate generation from citrate (reaction 15) and succinyl-CoA production from α-ketoglutarate (reaction 16) show little difference with the notable exception of the *Burn+Fasted* group (group D), where there is a significant jump in flux 16 relative to flux 15 (the ratios of flux 16 to flux 15 are 1.07, 0.98, 1.06, and 2.24 in groups A, B, C, and D, respectively) due to the increased contribution of glutamate and glutamine to α-ketoglutarate production (reactions 31 and 32) in that group. Taken together, these data show that in the sham groups, fasting increased TCA cycle (reactions 14–19) fluxes, which likely reflects the increased flux of pyruvate to oxaloacetate as a result of increased lactate uptake rate (reaction 12 in Figure 7) in the *Sham+Fasted* (group B) vs. the *Sham+Fed* (group A) groups. Conversely, in the burn groups, fasting increased oxaloacetate production from α-ketoglutarate (TCA cycle reactions 16–19), but not α-ketoglutarate generation from oxaloacetate (TCA cycle reactions 14–15), because in this case the additional carbon was supplied via the anaplerotic route represented where α-ketoglutarate was produced from glutamine and glutamate (reactions 31 and 32) in the *Burn+Fasted* group (group D). The rate of urea production from arginine (reaction 20), was also higher in the *Burn+Fasted* group (group D), consistent with the measured increase in urea output (Figure 3). Electron chain reactions (reactions 51 and 52 in Figure 6) were increased in the burn groups (groups C and D), which correlates with the increased TCA fluxes in *Burn+Fasted* group and increased fatty acid oxidation (reactions 46 and 47 in Figure 6) in the *Burn+Fed* group. In spite of the increased TCA fluxes in the *Sham+Fasted* (group B) group, the electron chain reactions were not elevated compared to the other groups, which might be the result of decreased fatty acid oxidation in that particular group.

**Figure 9:**
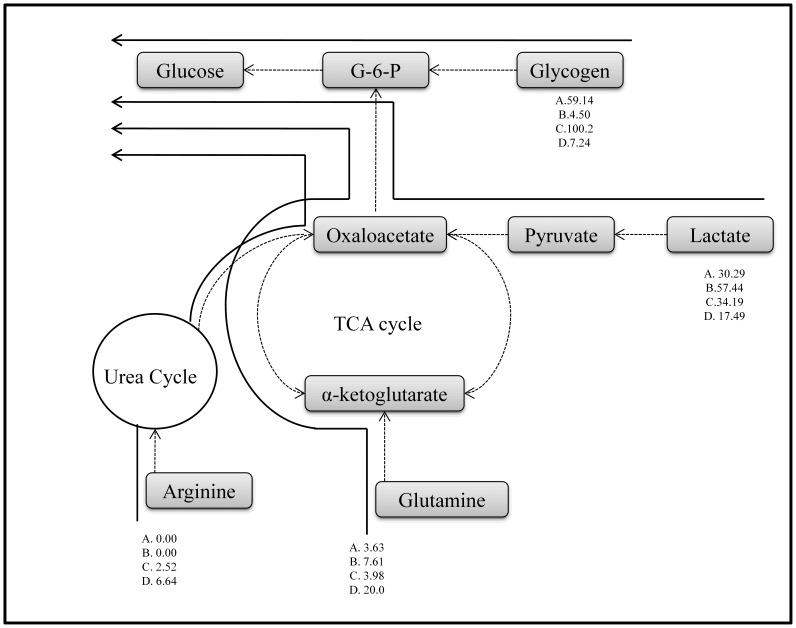
Detailed flux map showing elementary mode pathways for major potential gluconeogenic substrates. Flux values are expressed in terms of µmol/g liver/h. For example, in the *Sham+Fasted* group, 57.44 µmol/g liver/h lactate were utilized to produce glucose. A: Sham+Fed group; B: Sham+Fasted group; C: Burn+Fed group; D: Burn+Fasted group.

Since a weight for each pathway was assigned while solving the optimization problem, this can be used to quantitatively determine the contribution of major substrates to glucose production [31] as shown in Figure 9. In the *Sham+Fed* and *Burn+Fed* groups (groups A and C), glycogen breakdown contributed the largest amounts (59.14 µmol/g liver/h and 100.2 µmol/g liver/h, respectively), to glucose production. The next major contributor to glucose production was lactate (30.29 µmol/g liver/h and 34.19 µmol/g liver/h, respectively), while other sources – and in particular amino acids - were very small. In the fasted groups (groups B and D), glycogen contributions became very small as would be expected since the fasting protocol would be expected to deplete glycogen stores by the time livers are examined by perfusion [4], [34], [35]. In the *Sham+Fasted* group (group B), 57.44 µmol/g liver/h lactate (∼65% of total lactate uptake) were utilized for glucose production, and this value decreased to 17.49 µmol/g liver/h in the *Burn+Fasted* group (group D). Conversely, the usage of glutamine substrate for glucose production increased from 7.61 µmol/g liver/h glutamine in the *Sham+Fasted* group (group B), to 20.0 µmol/g liver/h glutamine (∼50% of total glutamine uptake) in the *Burn+Fasted* group (group D). The other major carbon source included arginine, which had no contribution in the sham groups (groups A and B), but some contribution in the burn groups (groups C and D). In the *Burn+Fasted* group (group D), 6.64 µmol/g liver/h arginine (∼20% of total arginine uptake) contributed to glucose production.

## Discussion

The objective of this study was to determine the effect of fasting on the hepatic response to burn injury in a rat model. Flux analysis determined that gluconeogenesis, glycogenolysis and fatty acid oxidation were active in all conditions, while the reverse pathways glycolysis, glycogen synthesis, and fatty acid synthesis were inhibited, even in the fed animals. While burn injury upregulated urea cycle fluxes in both fed and fasted groups, the impact on amino acid utilization was minimal in the fed group, but dramatic in the fasted group, with a significant increase in glycine, glutamine, arginine, and methionine uptake, with concomitant increase in ornithine release. The data also show the effect of burn injury on the fasting response of the liver, which was clearly different in sham-burned vs. burned animals. Overall, the main substrate for fasting-induced gluconeogenesis switched from lactate in sham-burned animals to amino acids (and more specifically glutamine and arginine) in burned animals. Taken together, these results suggest that the fed state prevents the burn-induced increase in hepatic amino acid utilization for gluconeogenesis. A major difference between fed and fasted states is the presence of glycogen stores in the former case and lack thereof in the latter. Thus, strategies that increase and/or maintain internal sources of glucose should be investigated to see if they can prevent increased hepatic amino acid utilization after burn injury.

The external fluxes which are experimentally determined correspond to the rates of metabolites that are secreted or taken up by the liver. How these rates are related to each other is a function of the internal metabolic network (which we represented by a stoichiometric model), which can reveal several contributions to a particular product. For example, urea and glutamate production (experimentally determined) were higher in the *Burn+Fed* group compared to the *Sham+Fed* group while glutamine, ornithine, and arginine fluxes were the same (Figures 2 and 3). Our model maximizing the activity of short pathways determined that production of glutamate from various amino acids such as glutamine, ornithine, proline and histidine (reactions 32–35) was higher in the *Burn+Fed* group compared to the *Sham+Fed* group (Table S5). Similarly, intracellular production of aspartate from oxaloacetate was higher in the *Burn+Fed* group compared to the *Sham+Fed* group (reaction 38, Table S5). Aspartate together with ammonium is one of the major sources for the arginine and urea production.

Several prior studies have reported on the response of isolated perfused livers (albeit in the absence of oxygen carriers) to a systemic burn injury similar to that applied in the current study evaluated under fasted conditions [5], [6], [8], [9]. The experimental data for fasted animals herein are largely consistent with those studies. Notably, oxygen and glutamine uptake, as well as urea production increased while net glucose output did not change. In a prior study where metabolites fluxes were measured in vivo in rats subjected to 20% and 40% TBSA burns [23], many of the changes mentioned above were seen in the larger burn group (40% TBSA) but not in the smaller burn (20% TBSA), even though the latter is a more similar injury model to that used herein. Possible explanations for the discrepancies include the fact that the perfused livers were not exposed to circulating factors (e.g. insulin, glucagon and other hormones) as well as substrate concentrations changes that occur in vivo. Therefore, further studies to examine the effect of these relevant stimuli are warranted. When considering the data obtained from fed animals, it is somewhat surprising that those animals did not show increased amino acid utilization after burns, when considering clinical evidence from burn patients, who are maintained in a fed state, suggesting the contrary. It is noteworthy that this study was limited to comparing “fasted” to “fed” using a particular feeding approach. It is possible that not all fed states are equal as the feeding strategy is likely to be important. The impact of feeding on the burn response could be further studied by using a diet that is more similar to the current parenteral feeding strategies that are used on patients.

The underlying mechanism for the increased reliance on amino acids for gluconeogenesis in the burned condition is unknown. The normal (i.e. in the absence of injury) response to fasting involves the breakdown of liver and skeletal muscle glycogen stores to release glucose into the circulation, as well as the use of lactate from the Cori cycle, in which case the utilization of other carbon sources is very limited [31]. On the other hand, the typical response to severe burn injury involves skeletal muscle protein breakdown and increased hepatic usage of liberated amino acids [36]. An analysis of hepatic fluxes measured in vivo in fasted burned rats concluded that livers extract amino acids more avidly than sham controls [23]. Consistent with these notions, it appears that the liver is actively switching from lactate to amino acids as substrate for fasting-induced gluconeogenesis. Among the amino acids found to be significantly affected in this study are glutamine and arginine, both of which have been reported to be either beneficial or “conditionally essential” after burn injury [7], [37], [38]. This is also consistent with prior findings of burn injury-induced increases in the expression of amino acid transporters for glutamine and arginine in the liver [8], [39], [40]. Glycine and methionine were also upregulated, and therefore may be potential candidates to further examine as nutritional supplements. Various nutritional options could be tested on animals using the procedures as described herein, where the liver metabolic responses from animals given different feeds (in terms of composition, dose, timing, and route of administration) could be compared.

In summary, we analyzed the effect of burn injury on the metabolism of livers isolated 4 days after burn injury in both fed and fasted animals. In fed animals, injury increased glucose output mainly from glycogen breakdown and minimally impacted amino acid metabolism. In fasted animals, injury did not increase glucose output but increased urea production and the uptake of several amino acids, namely glutamine, arginine, glycine, and methionine. Furthermore, sham-burn animals responded to fasting by triggering gluconeogenesis from lactate; however, in burned animals the preferred gluconeogenic substrate was amino acids. Taken together, these results suggest that the fed state prevents the burn-induced increase in hepatic amino acid utilization for gluconeogenesis. The possible role of glycogen stores and means to increase and/or maintain internal sources of glucose to prevent increased hepatic amino acid utilization warrant further study.

## Supporting Information

Table S1 **Substrate levels in fresh perfusate.**
(DOC)

Table S2 **Measured release rates of extracellular metabolites.**
(DOC)

Table S3 **The maximum and minimum values of measured extracellular fluxes used in metabolic model.**
(DOC)

Table S4 **Metabolites subject to mass balances.**
(DOC)

Table S5 **Calculated internal fluxes based on the assumption that short pathways tend to gain higher weight values.**
(DOC)

## References

[1] Mizock BA (1995) Alterations in Carbohydrate-Metabolism During Stress - a Review of the Literature. American Journal of Medicine 98: 75–84. 7825623 10.1016/S0002-9343(99)80083-7

[2] Wolfe RR, Herndon DN, Jahoor F, Miyoshi H, Wolfe M (1987) Effect of Severe Burn Injury on Substrate Cycling by Glucose and Fatty-Acids. New England Journal of Medicine 317: 403–408. 3614284 10.1056/NEJM198708133170702

[3] Bessey PQ, Jiang ZM, Johnson DJ, Smith RJ, Wilmore DW (1989) Posttraumatic Skeletal-Muscle Proteolysis - the Role of the Hormonal Environment. World Journal of Surgery 13: 465–471. 2672616 10.1007/BF01660758

[4] Yamaguchi Y, Yu YM, Zupke C, Yarmush DM, Berthiaume F, et al (1997) Effect of burn injury on glucose and nitrogen metabolism in the liver: preliminary studies in a perfused liver system. Surgery 121: 295–303. 9092130 10.1016/s0039-6060(97)90358-5

[5] Lee K, Berthiaume F, Stephanopoulos GN, Yarmush DM, Yarmush ML (2000) Metabolic flux analysis of post-burn hepatic hypermetabolism. Metabol Eng 2: 312–327. 10.1006/mben.2000.0160 11120643

[6] Lee K, Berthiaume F, Stephanopoulos GN, Yarmush ML (2003) Profiling of dynamic changes in hypermetabolic livers. Biotechnol Bioeng 83: 400–415. 12800135 10.1002/bit.10682

[7] Chen CL, Fei Z, Carter EA, Lu XM, Hu RH, et al (2003) Metabolic fate of extrahepatic arginine in liver after burn injury. Metabolism 52: 1232–1239. 14564672 10.1016/s0026-0495(03)00282-8

[8] Banta S, Vemula M, Yokoyama T, Jayaraman A, Berthiaume F, et al (2007) Contribution of gene expression to metabolic fluxes in hypermetabolic livers induced through burn injury and cecal ligation and puncture in rats. Biotechnol Bioeng 97: 118–137. 17009336 10.1002/bit.21200 PMC3199956

[9] Banta S, Yokoyama T, Berthiaume F, Yarmush ML (2005) Effects of dehydroepiandrosterone administration on rat hepatic metabolism following thermal injury. J Surg Res 127: 93–105. 15882877 10.1016/j.jss.2005.01.001

[10] Yarmush DM, MacDonald AD, Foy BD, Berthiaume F, Tompkins RG, et al (1999) Cutaneous burn injury alters relative tricarboxylic acid cycle fluxes in rat liver. J Burn Care Rehabil 20: 292–302. 10425591 10.1097/00004630-199907000-00004

[11] Hu RH, Yu YM, Costa D, Young VR, Ryan CM, et al (1998) A rabbit model for metabolic studies after burn injury. J Surg Res 75: 153–160. 9655088 10.1006/jsre.1998.5274

[12] Herndon DN, Tompkins RG (2004) Support of the metabolic response to burn injury. Lancet 363: 1895–1902. 15183630 10.1016/S0140-6736(04)16360-5

[13] Jeschke MG, Herndon DN, Ebener C, Barrow RE, Jauch K-W (2001) Nutritional intervention high in vitamins, protein, amino acids, and w3 fatty acids improves protein metabolism during the hypermetabolic state after thermal injury. Arch Surg 136: 1301–1306. 11695977 10.1001/archsurg.136.11.1301

[14] Orman MA, Ierapetritou MG, Androulakis IP, Berthiaume F (2011) Metabolic response of perfused livers to various oxygenation conditions. Biotechnol Bioeng 108: 2947–2957. 21755498 10.1002/bit.23261 PMC3193557

[15] Orman MA, Androulakis IP, Berthiaume F, Ierapetritou MG (2012) Metabolic network analysis of perfused livers under fed and fasted states: incorporating thermodynamic and futile-cycle-associated regulatory constraints. J Theor Biol 293: 101–110. 22037644 10.1016/j.jtbi.2011.10.019

[16] Yang Q, Orman MA, Berthiaume F, Ierapetritou MG, Androulakis IP (2011) Dynamics of Short-Term Gene Expression Profiling in Liver Following Thermal Injury. J Surg Res. 10.1016/j.jss.2011.09.052 PMC3319855 22099593

[17] Walker HL, Mason AD Jr (1968) A standard animal burn. J Trauma 8: 1049–1051. 5722120 10.1097/00005373-196811000-00006

[18] Herndon DN, Wilmore DW, Mason AD Jr (1978) Development and analysis of a small animal model simulating the human postburn hypermetabolic response. J Surg Res 25: 394–403. 713539 10.1016/s0022-4804(78)80003-1

[19] Ovacik MA, Sukumaran S, Almon RR, DuBois DC, Jusko WJ, et al (2010) Circadian signatures in rat liver: from gene expression to pathways. BMC Bioinformatics 11: 540. 21040584 10.1186/1471-2105-11-540 PMC2990769

[20] Nguyen TT, Almon RR, DuBois DC, Jusko WJ, Androulakis IP (2010) Importance of replication in analyzing time-series gene expression data: corticosteroid dynamics and circadian patterns in rat liver. BMC Bioinformatics 11: 279. 20500897 10.1186/1471-2105-11-279 PMC2889936

[21] Almon RR, Yang E, Lai W, Androulakis IP, DuBois DC, et al (2008) Circadian variations in rat liver gene expression: relationships to drug actions. J Pharmacol Exp Ther 326: 700–716. 18562560 10.1124/jpet.108.140186 PMC2561907

[22] Mortimore GE, Surmacz CA (1984) Liver perfusion: an in vitro technique for the study of intracellular protein turnover and its regulation in vivo. Proc Nutr Soc 43: 161–177. 6382268 10.1079/pns19840039

[23] Izamis ML, Sharma NS, Uygun B, Bieganski R, Saeidi N, et al (2011) In situ metabolic flux analysis to quantify the liver metabolic response to experimental burn injury. Biotechnol Bioeng 108: 839–852. 21404258 10.1002/bit.22998 PMC3277812

[24] Chan C, Berthiaume F, Lee K, Yarmush ML (2003) Metabolic flux analysis of cultured hepatocytes exposed to plasma. Biotechnology and Bioengineering 81: 33–49. 12432579 10.1002/bit.10453

[25] Chan C, Berthiaume F, Lee K, Yarmush ML (2003) Metabolic flux analysis of hepatocyte function in hormone- and amino acid-supplemented plasma. Metabolic Engineering 5: 1–15. 12749840 10.1016/s1096-7176(02)00011-3

[26] Nolan RP, Fenley AP, Lee K (2006) Identification of distributed metabolic objectives in the hypermetabolic liver by flux and energy balance analysis. Metab Eng 8: 30–45. 16289779 10.1016/j.ymben.2005.08.004

[27] Orman MA, Arai K, Yarmush ML, Androulakis IP, Berthiaume F, et al (2010) Metabolic flux determination in perfused livers by mass balance analysis: effect of fasting. Biotechnol Bioeng 107: 825–835. 20661905 10.1002/bit.22878

[28] Arai K, Lee K, Berthiaume F, Tompkins RG, Yarmush ML (2001) Intrahepatic amino acid and glucose metabolism in a D-galactosamine-induced rat liver failure model. Hepatology 34: 360–371. 11481621 10.1053/jhep.2001.26515

[29] Yang H, Roth CM, Ierapetritou MG (2011) Analysis of amino acid supplementation effects on hepatocyte cultures using flux balance analysis. OMICS 15: 449–460. 21410329 10.1089/omi.2010.0070

[30] Klamt S, Saez-Rodriguez J, Gilles ED (2007) Structural and functional analysis of cellular networks with CellNetAnalyzer. BMC Syst Biol 1: 2. 17408509 10.1186/1752-0509-1-2 PMC1847467

[31] Orman MA, Berthiaume F, Androulakis IP, Ierapetritou MG (2011) Pathway analysis of liver metabolism under stressed condition. J Theor Biol 272: 131–140. 21163266 10.1016/j.jtbi.2010.11.042 PMC3038651

[32] Schwarz R, Musch P, von Kamp A, Engels B, Schirmer H, et al (2005) YANA - a software tool for analyzing flux modes, gene-expression and enzyme activities. BMC Bioinformatics 6: 135. 15929789 10.1186/1471-2105-6-135 PMC1175843

[33] Rutter MT, Zufall RA (2004) Pathway length and evolutionary constraint in amino acid biosynthesis. J Mol Evol 58: 218–224. 15042343 10.1007/s00239-003-2546-y

[34] Soares AF, Carvalho RA, Veiga FJ, Jones JG (2010) Effects of galactose on direct and indirect pathway estimates of hepatic glycogen synthesis. Metab Eng 12: 552–560. 20797446 10.1016/j.ymben.2010.08.002

[35] Soares AF, Viega FJ, Carvalho RA, Jones JG (2009) Quantifying hepatic glycogen synthesis by direct and indirect pathways in rats under normal ad libitum feeding conditions. Magn Reson Med 61: 1–5. 19097206 10.1002/mrm.21830

[36] Biolo G, Toigo G, Ciocchi B, Situlin R, Iscra F, et al (1997) Metabolic response to injury and sepsis: changes in protein metabolism. Nutrition 13: 52–57. 10.1016/s0899-9007(97)00206-2 9290110

[37] Wilmore DW (2001) The effect of glutamine supplementation in patients following elective surgery and accidental injury. J Nutr 131: 2543S–2549S; discussion 2550S–2541S. 10.1093/jn/131.9.2543S 11533310

[38] Yu YM, Ryan CM, Castillo L, Lu XM, Beaumier L, et al (2001) Arginine and ornithine kinetics in severely burned patients: increased rate of arginine disposal. Am J Physiol Endocrinol Metab 280: E509–517. 11171607 10.1152/ajpendo.2001.280.3.E509

[39] Vemula M, Berthiaume F, Jayaraman A, Yarmush ML (2004) Expression profiling analysis of the metabolic and inflammatory changes following burn injury in rats. Physiological Genomics 18: 87–98. 15114001 10.1152/physiolgenomics.00189.2003

[40] Lohmann R, Souba WW, Bode BP (1999) Rat liver endothelial cell glutamine transporter and glutaminase expression contrast with parenchymal cells. Am J Physiol 276: G743–G750. 10070052 10.1152/ajpgi.1999.276.3.G743

