# Supplementary material for: Effect of Fasting on the Metabolic Response of Liver to Experimental Burn Injury
Source: PLoS One. 2013 Feb 5;8(2):e54825. doi: 10.1371/journal.pone.0054825 (PMC3564862; doi:10.1371/journal.pone.0054825)
Supplement: Table S1 — Substrate levels in fresh perfusate. (DOC) [file pone.0054825.s001.doc]

**Table S1.** Substrate levels in fresh perfusate (μmol/100 ml).

| Alanine | 48.4 |
| --- | --- |
| Arginine | 72.3 |
| Asparagine | 78 |
| Aspartic acid | 6.3 |
| Cysteine | 25.8 |
| Glutamic acid | 32.8 |
| Glutamine | 199.8 |
| Glycine | 38 |
| Histidine | 27.1 |
| Isoleucine | 39.6 |
| Leucine | 39.6 |
| Lysine | 49.6 |
| Methionine | 10.1 |
| Phenylalanine | 19.4 |
| Proline | 41.6 |
| Serine | 63.2 |
| Threonine | 40.2 |
| Tryptophan | 4.9 |
| Tyrosine | 28.6 |
| Valine | 39.3 |
| Glucose | 555.5 |
| Lactic acid | 500 |
| Sodium pyruvate | 50 |
| Sodium bicarbonate | 2618.7 |
