# Supplementary material for: Effect of Fasting on the Metabolic Response of Liver to Experimental Burn Injury
Source: PLoS One. 2013 Feb 5;8(2):e54825. doi: 10.1371/journal.pone.0054825 (PMC3564862; doi:10.1371/journal.pone.0054825)
Supplement: Table S2 — Measured release rates of extracellular metabolites. (DOC) [file pone.0054825.s002.doc]

**Table S2**. Measured release rates of extracellular metabolites (μmol/g liver/h).

|  | Sham+Fed | Sham+Fasted | Burn+Fed | Burn+Fasted |
| --- | --- | --- | --- | --- |
| Aspartate | 0.287±0.35 | 0.282±0.27 | 0.054±1.35 | 0.499±0.44 |
| Glycine | -2.88±1.21 | -1.1±2.22 | -6.43±0.62 | -8.45±1.26 |
| Histidine | -2.55±0.97 | -1.15±1.62 | -3.68±0.98 | -5.14±0.72 |
| Ammonia | -0.85±3.21 | -5.35±1.06 | -3.07±2.45 | 0.668±1.74 |
| Arginine | -4.36±2.38 | -5.69±4.27 | -9.36±3.08 | -30±7.82 |
| Threonine | -1.08±3.04 | 2.743±3.93 | -0.27±1.96 | -4.79±0.49 |
| Alanine | -2.43±1.07 | 2.5±1.99 | -1.8±2.16 | -2.87±0.89 |
| Proline | -1.71±0.59 | 1.696±4.25 | -2.39±1.67 | -4.32±0.48 |
| Tyrosine | -0.09±0.55 | -2.9±9.74 | 1.126±0.29 | 0.071±0.44 |
| Valine | 2.033±0.17 | 3.515±3.08 | 3.087±2.28 | 1.892±1.37 |
| Methionine | -0.72±0.28 | -0.32±0.73 | -0.96±0.07 | -1.87±0.29 |
| Lysine | 0.446±1.77 | 2.6±2.37 | -3.68±11.56 | 3.906±6.47 |
| Isoleucine | 0.527±0.52 | 2.635±3.15 | 1.512±2.29 | 1.322±1.47 |
| Leucine | 2.612±0.75 | 4.932±3.74 | 4.28±1.76 | 2.219±1.64 |
| Phenylalanine | -2.86±1.00 | -1.74±1.77 | -2.99±0.71 | -3.24±0.20 |
| Glutamic acid | 5.104±2.05 | 7.914±7.74 | 10.92±1.11 | 12.75±5.68 |
| Glutamine | -12.7±4.85 | -15.9±5.54 | -10.8±3.27 | -45.6±4.00 |
| Ornithine | 0.802±0.27 | 2.176±1.30 | 1.482±0.83 | 8.497±1.75 |
| Glucose | 115.5±13.06 | 69.76±22.21 | 163.8±19.81 | 57.13±9.21 |
| Lactate | -15.6±17.55 | -100±17.16 | -16.3±18.02 | -5.55±16.75 |
| Urea | 17.14±1.68 | 10.94±2.33 | 29.04±1.26 | 38.43±6.74 |
| β-hydroxybutyrate | 58.61±2.30 | 76.77±7.11 | 69.21±3.87 | 71.83±3.20 |
| Oxygen | -316±14.27 | -321±19.34 | -369±36.04 | -378±31.63 |

Data shown are means±SD. N≥3. A negative value indicates net uptake.
