# Supplementary material for: Effect of Fasting on the Metabolic Response of Liver to Experimental Burn Injury
Source: PLoS One. 2013 Feb 5;8(2):e54825. doi: 10.1371/journal.pone.0054825 (PMC3564862; doi:10.1371/journal.pone.0054825)
Supplement: Table S3 — The maximum and minimum values of measured extracellular fluxes used in metabolic model. (DOC) [file pone.0054825.s003.doc]

**Table S3**. The maximum and minimum values of measured extracellular fluxes used in metabolic model (μmol/g liver/h).

| **Metabolites** | **Sham+Fed** | | **Sham+Fasted** | | **Burn+Fed** | | **Burn+Fasted** | |
| --- | --- | --- | --- | --- | --- | --- | --- | --- |
|  | ***Max.*** | ***Min.*** | ***Max.*** | ***Min.*** | ***Max.*** | ***Min.*** | ***Max.*** | ***Min.*** |
| Aspartate | -0.13 | 0.74 | -0.05 | 0.60 | -1.85 | 1.35 | 0.04 | 1.10 |
| Glycine | -4.09 | -1.22 | -4.14 | 1.10 | -7.14 | -5.67 | -9.39 | -6.60 |
| Histidine | -3.88 | -1.60 | -3.42 | 0.28 | -4.90 | -2.51 | -6.08 | -4.32 |
| Ammonia | -5.33 | 2.05 | -6.67 | -4.07 | -6.45 | -0.61 | -0.85 | 3.18 |
| Arginine | -6.14 | -0.99 | -10.18 | 0.05 | -13.70 | -6.74 | -39.52 | -21.09 |
| Threonine | -5.30 | 1.71 | -2.25 | 7.36 | -2.09 | 2.47 | -5.36 | -4.21 |
| Alanine | -3.41 | -0.94 | -0.27 | 4.34 | -4.97 | -0.36 | -3.76 | -1.84 |
| Proline | -2.47 | -1.02 | -4.30 | 5.03 | -4.55 | -0.53 | -4.69 | -3.66 |
| Tyrosine | -0.81 | 0.53 | -16.56 | 5.44 | 0.72 | 1.39 | -0.41 | 0.65 |
| Valine | 1.85 | 2.26 | -0.59 | 6.84 | 0.67 | 5.34 | 0.85 | 3.80 |
| Methionine | -1.12 | -0.51 | -1.20 | 0.59 | -1.07 | -0.90 | -2.18 | -1.49 |
| Lysine | -1.84 | 2.49 | -0.53 | 5.20 | -20.66 | 5.16 | -2.65 | 12.74 |
| Isoleucine | -0.10 | 1.17 | -1.68 | 5.77 | -1.49 | 3.72 | -0.16 | 3.26 |
| Leucine | 1.73 | 3.58 | 0.12 | 9.24 | 2.26 | 5.90 | 0.26 | 4.25 |
| Phenylalanine | -3.86 | -1.49 | -3.87 | 0.48 | -4.05 | -2.56 | -3.43 | -2.96 |
| Glutamic acid | 2.65 | 7.68 | -2.27 | 16.47 | 9.57 | 11.88 | 7.59 | 17.66 |
| Glutamine | -18.83 | -6.96 | -23.71 | -11.92 | -15.09 | -7.20 | -48.86 | -40.78 |
| Ornithine | 0.48 | 1.15 | 0.35 | 3.26 | 0.83 | 2.57 | 6.97 | 10.28 |
| Glucose | 97.09 | 125.77 | 49.47 | 100.67 | 141.37 | 186.77 | 46.64 | 69.11 |
| Lactate | -34.06 | 8.00 | -124.53 | -87.00 | -38.42 | 4.12 | -21.54 | 8.92 |
| Urea | 15.03 | 19.13 | 9.02 | 14.22 | 27.61 | 30.48 | 28.40 | 42.97 |
| β-hydroxybutyrate | 55.68 | 62.73 | 66.75 | 82.60 | 65.14 | 73.62 | 68.00 | 74.48 |
| Oxygen | -329.29 | -296.40 | -343.64 | -296.40 | -393.22 | -315.55 | -424.10 | -352.01 |
