# Supplementary material for: Effect of Fasting on the Metabolic Response of Liver to Experimental Burn Injury
Source: PLoS One. 2013 Feb 5;8(2):e54825. doi: 10.1371/journal.pone.0054825 (PMC3564862; doi:10.1371/journal.pone.0054825)
Supplement: Table S4 — Metabolites subject to mass balances. (DOC) [file pone.0054825.s004.doc]

**Table S4.** Metabolites subject to mass balances.

| Acetoacetate | Glutamine | Phenylalanine |
| --- | --- | --- |
| Acetoacetyl-CoA | Glyceraldehyde-3-P | Proline |
| Acetyl-CoA | Glycine | Propinoyl-CoA |
| α-ketoglutarate | Glycogen | Pyruvate |
| Alanine | Glycogen(n-1) | Serine |
| Arginine | Histidine | Succinyl-CoA |
| Asparagine | Isoleucine | Threonine |
| Aspartate | Lactate | Tyrosine |
| β-OH-butyrate | Leucine | Urea |
| Citrate | Lysine | Valine |
| Citrulline | Malate |  |
| CO_2_ | Methionine |  |
| Cysteine | NADH |  |
| FADH_2_ | NH_4_^+^ |  |
| Fructose-1,6-P_2_ | O_2_ |  |
| Fructose-6-P | Ornithine |  |
| Fumarate | Oxaloacetate |  |
| Glucose | Palmitate |  |
| Glucose-6-P | Palmitoylglycerol |  |
| Glutamate | PEP |  |
