# Supplementary material for: Effect of Fasting on the Metabolic Response of Liver to Experimental Burn Injury
Source: PLoS One. 2013 Feb 5;8(2):e54825. doi: 10.1371/journal.pone.0054825 (PMC3564862; doi:10.1371/journal.pone.0054825)
Supplement: Table S5 — Calculated internal fluxes based on the assumption that short pathways tend to gain higher weight values. (DOC) [file pone.0054825.s005.doc]

**Table S5.** Calculated internal fluxes based on the assumption that short pathways tend to gain higher weight values (μmol/g liver/h).

| **Reactions** | **Sham+Fed** | **Sham+Fasted** | **Burn+Fed** | **Burn+Fasted** |
| --- | --- | --- | --- | --- |
| Reaction 1 | 97.09006 | 49.47162 | 141.3728 | 46.63661 |
| Reaction 2 | 38.96009 | 50.29737 | 42.31264 | 49.54019 |
| Reaction 3 | 38.96009 | 50.29737 | 42.31264 | 49.54019 |
| Reaction 4 | 38.96009 | 50.29737 | 42.31264 | 49.54019 |
| Reaction 5 | 63.22996 | 92.55008 | 68.56164 | 86.29094 |
| Reaction 6 | 63.22996 | 92.55008 | 68.56164 | 86.29094 |
| Reaction 7 | 59.33497 | 82.44667 | 65.13614 | 35.14068 |
| Reaction 8 | 0.000 | 0.000 | 0.000 | 0.000 |
| Reaction 9 | 0.000 | 0.000 | 0.000 | 0.000 |
| Reaction 10 | 0.000 | 0.000 | 0.000 | 0.000 |
| Reaction 11 | 1.015993 | 5.343482 | 1.174017 | 10.14878 |
| Reaction 12 | 34.05924 | 87.00151 | 38.42355 | 21.53568 |
| Reaction 13 | 0.000 | 1.794119 | 0.000 | 3.165532 |
| Reaction 14 | 29.11962 | 71.09103 | 40.6345 | 36.42828 |
| Reaction 15 | 29.11962 | 71.09103 | 40.6345 | 36.42828 |
| Reaction 16 | 31.01869 | 69.74631 | 43.17146 | 81.64344 |
| Reaction 17 | 32.79486 | 73.22255 | 44.24145 | 84.83587 |
| Reaction 18 | 51.89743 | 94.21427 | 69.14748 | 110.555 |
| Reaction 19 | 51.89743 | 94.21427 | 69.14748 | 110.555 |
| Reaction 20 | 19.13411 | 14.22258 | 30.47688 | 42.96515 |
| Reaction 21 | 18.14447 | 13.87597 | 23.7343 | 21.87999 |
| Reaction 22 | 18.14447 | 13.87597 | 23.7343 | 21.87999 |
| Reaction 23 | 2.158272 | -4.34361 | 0.742628 | 1.8358 |
| Reaction 24 | 19.48652 | 19.37346 | 21.76681 | 23.79534 |
| Reaction 25 | 3.630934 | -17.7906 | 4.203152 | -8.8606 |
| Reaction 26 | 0.000 | 2.253013 | 0.000 | 5.355879 |
| Reaction 27 | 0.609033 | 0.574633 | 2.836797 | 5.975599 |
| Reaction 28 | 0.000 | 0.533805 | 0.000 | 1.39E-05 |
| Reaction 29 | 1.490946 | 3.401863 | 2.563154 | 3.428026 |
| Reaction 30 | 0.958093 | 7.115744 | 1.171732 | 3.839165 |
| Reaction 31 | 1.899069 | -1.34471 | 2.536961 | 45.21515 |
| Reaction 32 | 6.963101 | 11.92297 | 7.201661 | 40.77935 |
| Reaction 33 | 0.000 | 0.000 | 4.171006 | 14.11692 |
| Reaction 34 | 1.019607 | 2.003874 | 0.534859 | 3.656431 |
| Reaction 35 | 1.596377 | 1.199877 | 2.513503 | 4.324443 |
| Reaction 36 | 1.122512 | 1.201173 | 1.069984 | 2.18026 |
| Reaction 37 | 1.776174 | 3.47624 | 1.069984 | 3.192433 |
| Reaction 38 | -18.8828 | -13.0198 | -25.0875 | -22.9765 |
| Reaction 39 | 0.000 | 1.458832 | 0.000 | 1.54E-05 |
| Reaction 40 | 0.554392 | 0.592054 | 0.000 | 0.85001 |
| Reaction 41 | 0.099271 | 1.683014 | 0.000 | 0.162164 |
| Reaction 42 | 0.000 | 0.117482 | 0.000 | 0.212245 |
| Reaction 43 | 14.69022 | 8.044655 | 16.06363 | 12.78945 |
| Reaction 44 | 44.07066 | 24.13397 | 48.19089 | 38.36836 |
| Reaction 45 | 0.000 | 0.000 | 0.000 | 0.000 |
| Reaction 46 | 161.7725 | 63.91416 | 172.4463 | 139.7072 |
| Reaction 47 | 161.7725 | 64.44797 | 172.4463 | 139.7072 |
| Reaction 48 | 62.73057 | 82.60361 | 73.61802 | 74.47675 |
| Reaction 49 | 0.000 | 0.000 | 0.000 | 0.000 |
| Reaction 50 | 59.14596 | 4.517733 | 100.2341 | 7.245196 |
| Reaction 51 | 337.5127 | 310.4487 | 394.5094 | 426.137 |
| Reaction 52 | 341.9432 | 245.0867 | 381.5776 | 354.6388 |

**Note:** Glycolytic reactions (Rxns 8, 9, 10), fatty acid synthesis (Rxn 45), and glycogen synthesis (Rxn 49) which form futile cycles were found to be inactive. Fluxes of reactions related to amino acid metabolism (Rxn23 –Rxn 42) were found to be smaller compared to gluconeogenic, TCA and fatty acid oxidation fluxes.
